# Projections for fast protein structure retrieval

**DOI:** 10.1186/1471-2105-7-S5-S5

**Published:** 2006-12-18

**Authors:** Sourangshu Bhattacharya, Chiranjib Bhattacharyya, Nagasuma R Chandra

**Affiliations:** 1 Dept. of Computer Science and Automation, Indian Institute of Science, Bangalore – 560012, India; 2 Bioinformatics Center, Indian Institute of Science, Bangalore – 560012, India

## Abstract

**Background:**

In recent times, there has been an exponential rise in the number of protein structures in databases e.g. PDB. So, design of fast algorithms capable of querying such databases is becoming an increasingly important research issue. This paper reports an algorithm, motivated from spectral graph matching techniques, for retrieving protein structures similar to a query structure from a large protein structure database. Each protein structure is specified by the 3D coordinates of residues of the protein. The algorithm is based on a novel characterization of the residues, called projections, leading to a similarity measure between the residues of the two proteins. This measure is exploited to efficiently compute the optimal equivalences.

**Results:**

Experimental results show that, the current algorithm outperforms the state of the art on benchmark datasets in terms of speed without losing accuracy. Search results on SCOP 95% nonredundant database, for fold similarity with 5 proteins from different SCOP classes show that the current method performs competitively with the standard algorithm CE. The algorithm is also capable of detecting non-topological similarities between two proteins which is not possible with most of the state of the art tools like Dali.

## Background

Retrieval of similar proteins from a database is a fundamental problem in Bioinformatics. Traditionally, similarity is defined in terms of scores of optimal sequence alignment between the amino acid sequences of the two proteins involved. However, it is well known that structures of proteins relate more accurately to their functions and evolutionary history than amino acid sequences. Thus, retrieval of structurally similar proteins becomes an important problem.

There has been a very rapid growth in the number of structures in protein databases in the past few years, with PDB [1] having more than 32000 structures (about 24 GB of data). Very fast algorithms are required for searching through such a huge amount of data for similar proteins. Many protein structure comparison algorithms have been proposed for finding out the extent of similarity between two proteins.  Unfortunately, the formulations have turned out to be NP-Hard [2].

Many heuristics have been proposed for comparing protein structures, e.g. Dali [3,4], C-alpha match [5], LOCK [6], SSAP [7], etc. Two main issues about protein structure comparison algorithms are:

• To what extent are extra atoms (those that are not present in the other structure) called *indels *tolerated.

• Whether *non-topological *similarities (those not following sequence order of the protein) are detected.

Unfortunately, most of the above mentioned algorithms are very slow for searching through databases of size similar to PDB. CE [8] was designed to search through PDB, and is very fast. However, it can't detect non-topological similarities. This is a limitation as many types of non-topological similarities (e.g. circular permutations [9-11]) are known to exist in nature. Some algorithms, e.g. [5] and [12] are capable of detecting non-topological similarities.

The aim in this paper, is to derive an alternate formulation of the protein structure comparison problem, resulting in development of a fast algorithm for comparing protein structures, called Matchprot. Moreover, this algorithm is capable of detecting non-topological similarities. Our formulation is motivated from the distance matrix overlap formulation [3]. A novel characterization of the residues of a protein in the context of its overall structure is calculated by projecting them on the real line in a neighborhood preserving way. This characterization is used to define a similarity function between the residues of two proteins and find the optimal equivalences. Our method is closely related to the Umeyama's method of matching weighted graphs [13] which uses eigenvectors of adjacency matrices.

Speedup in the computation of alignment is achieved by using the similarity function between residues from two structures, as opposed to similarity function on pairs of residues defined by the distance matrix formulation. Such an attempt has been made in SSAP [7], by using two level dynamic programming (DP). However, no single representation of each residue is obtained, leading to similarity scores with conflicting residue equivalences. Moreover, for every pair of residues a DP has to be run, making the program very slow. The current algorithm gives a single projection of the residues on the real line using eigenvector decomposition, for which highly efficient algorithms are known. Also, methods described in [6] and [14] use an initial secondary structure alignment to derive similarity score between residues. The current algorithm takes only tertiary structure into account and computes the alignment in one step, not iteratively.

The algorithm was tested extensively using 2 comprehensive benchmark datasets. The results obtained on benchmark datasets are comparable to those obtained with Dali, a state of the art protein structure comparison algorithm. Moreover, the algorithm was used to query a database of about 10000 protein structures (about 9 GB of data), for 5 different query proteins from the different protein classes. Results of this search, compared to the standard database search algorithm CE, are encouraging. Since the residues are characterized in the context of the whole structure, indels in the structures affect the actual characterization values. However, experimental results show that, in case of high structural similarity, correct equivalences are retrieved for upto 40% indels. Experimental results also confirm the algorithm's ability to detect non-topological similarities. Timing tests show that the algorithm is 2–3 times faster than CE, and 15–20 times faster than Dali.

## Results

A comprehensive set of experiments were performed in order to evaluate the fitness of Matchprot for protein structure retrieval and to ascertain the possible applications of such a retrieval tool. For this purpose, we implemented the algorithm on GCC/GNU-Linux. LAPACK [15] was used for eigenvector computations. Rasmol [16] was used for visualization of the structural superpositions and generating the images presented in this paper.

Firstly, in order to assess the quality of alignments generated by Matchprot vis-a-vis the standard protein structure comparison algorithm Dali, we run both the algorithms on two benchmark datasets: Fischer's [17] and Novotny et al.'s [18]. These datasets have over 200 pairs of similar protein structures from a varied class of proteins, which are "difficult" to detect. In the next subsection, we report detailed results from Matchprot and benchmark it against DALI.

Circular permutations are a specific type of non-topological similarity observed in nature [9,10], having many important applications. In the section titled Nontopological similarities we explore the ability of Matchprot for detecting non-topological similarities. The alignment, for a well known circularly permuted protein pair is reported. Next, in order to test Matchprot's performance in presence indels, we use it to compare domains of multi-domain proteins with with the full protein.

Structural Classification of Proteins (SCOP) [19] is a manually curated hierarchical classification system of proteins having about 70000 domains. Domains are classified at 4 levels of similarity: class, fold, superfamily and family. The level of similarity between two proteins is gives a lot of insight into their functions. In the next experiment (see the following section on SCOP), we searched SCOP 95% non-redundant dataset having about 10000 domains for 5 randomly selected query proteins using Matchprot, making a total of 50000 pairwise comparisons. We also compared the results at the fold level of similarity with that of the standard program CE.

Finally, we compare the running times taken by Matchprot, CE and Dali for proteins of different sizes. It clearly shows that matchprot is much faster than Dali and significantly faster than CE.

### Validation using Fischer's and Novotny *et al*.'s dataset

In order to assess the performance of Matchprot on benchmark datasets, we ran Matchprot on Fischer's [17] and Novotny et al.'s [18] dataset, and compared the results with those from Dali. Fischer's data set in a well known benchmark, and contains many difficult similarities. Novotny et al. have recently compared 11 fold recognition programs using their dataset.

There are 4 parameters to Matchprot, dimension of representation *k*, parameter for calculation of nearness matrix *α*, threshold for the similarity function *T*, and gap penalty *g*. In absence a clearly best set of values for parameters, Matchprot was run for 72 combinations of parameters, making a total of 4896 pairwise comparisons. Total time (user + system) taken by Matchprot was 27 min 38.58 s, whereas Dali took 52 min 8.21 s. The range of parameter values over which search was made is: *k *: {1, 3}, *α*: {5, 10, 15, 20}, *T *: {0.2, 0.5, 0.8}, and *g*: {0.1, 0.4, 0.7}.

Table 1 reports a summary of results from the experiment. The alignments were compared on the basis of RMSD and length of alignment. An alignment is said to be better than another (between the same pair), if the former has a higher length and lower RMSD than the later. Two alignments are called *level *if one has higher RMSD and higher length than the other.

Z-score measures the statistical significance of an alignment. However, it was noted that the Z-scores computed by the Matchprot and Dali were different even in case of similar alignments. For this reason, we chose to use RMSD and length of alignment, rather than Z-score for comparing alignments between Dali and Matchprot. The test on the benchmark data set shows that the quality of alignments given by Dali and Matchprot are comparable.

### Non-topological similarities, multiple domains and proteins with internal repeats

**Figure 1:**
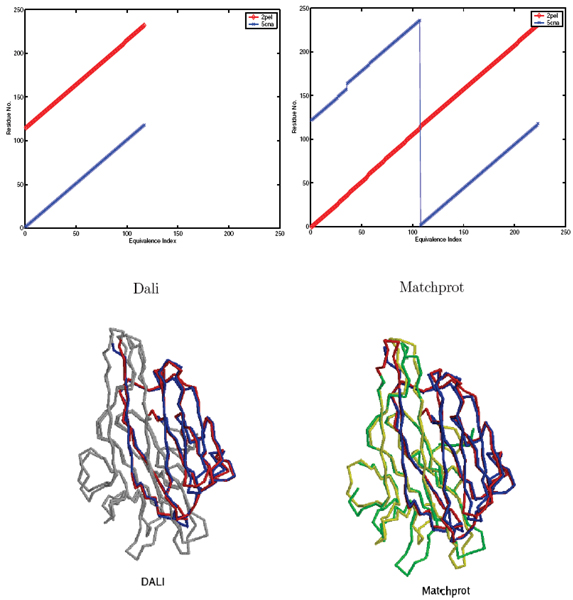
2pelA – 5cnaA alignments generated by Dali and Matchprot.

In order to demonstrate the performance of Matchprot in detecting non-topological similarities, we show the alignment of the famous circularly permuted pair 2pelA-5cnaA in detail. Figure 1 shows the alignment graphs for 2pelA-5cnaA with Y-axis showing the residue number as they appear in the chain. The negative jump in alignment given by Matchprot indicates a circular permutation.

For 2pelA-5cnaA, Matchprot gives a 223 residue alignment with RMSD 1.48Å whereas Dali gives a 117 residue alignment with RMSD 1.3Å. The superpositions generated by the 2 programs are also shown. The equivalenced portions are shown in color combination *blue *↔ *red *and *yellow *↔ *green*. It is very clear that Dali detects only a portion of the total alignment.

Next, to test the extent to which Matchprot can handle indels in the structures, we compared 2 multi-domain proteins (each having 3 separate domains), taken from Novotny et. al.'s article [18], with various partial structures of the same proteins, including the individual domains. Table 2 gives results from these experiments. The first six entries in the table report comparison of 2HCK with partial structures obtained by deleting 4, 8, 12, 20, 50, and 100 residues, respectively. It can be seen these deletions are handled properly by the current algorithm.

Next six entries in Table 2 give results from comparison of 2HCK and 2SRC with their individual domains. In each case, only the largest domain is detected properly, which indicates that 'indels' upto 160 residues in a 440 amino acid protein is tolerated quite well. The present implementation however is not suitable to detect very large domain deletions, inducing indels more than 40% of the size of the query structure. This, although a limitation in that sense does not hinder commonly required structural comparisons, where each indel is generally in the order of a few residues only. Even in cases where large domain deletions exist, Matchprot returns a close but not a perfect alignment, which can be used as a seed to tune the query to that of an appropriate size to the structure of interest to subsequently get a more accurate alignment, thus overcoming the minor limitation of not tolerating very large deletions at the outset. Moreover (see section on SCOP) even with this limitation, the current algorithm performs competitively with state of the art algorithms for structure retrieval from databases.

Finally, in order to ascertain whether the local alignment procedure is fooled by proteins having internal symmetry, we compared 6 proteins showing high degree of internal symmetry with their homologues. Table 3 report the results from these experiments vis-a-vis those from Dali. It is clear that Matchprot detects all the matches correctly.

We tested the alignments given by matchprot on more than 100 other protein structure pairs. The experiments suggest that in most cases, Matchprot performs comparably with Dali.

### Structure based search on 5 SCOP classes

In order to judge Matchprot's performance in retrieving structurally similar proteins from databases, we searched the SCOP 95% non-redundant database, for similarities with 5 randomly selected SCOP domains (d101m__, d1htia_, d1jzba_, d2pela_, d7rsa__) from the 5 major SCOP classes. The ASTRAL (a derived dataset from SCOP) [20] 95% non-redundant dataset has about 10000 domains, occupying about 9 GB of disk space. Thus, about 50000 pairwise comparisons were performed. Since, the computation was performed on shared machines, reporting exact timing of the experiment is not possible. The computation takes roughly 1 day time on a 2.4 GHz mahcine.

Table 4 reports a summary of the search performed on 5 randomly selected domains. For each domain that is searched for, and for 3 levels of structural similarity (fold, superfamily & family), we report the number of similar structures in the database, the number of similar structures detected by Matchprot, and the number of false positives. In absence of other measures, the similarity was ascertained using a cuto3 on the Z-score. Accuracy of classification for a large number Z-score cutoffs were observed, and the best range of cutoffs were determined to be 2 – 6 for fold classification. It can be seen that Matchprot detects all structures having family level similarity and most of the structures having superfamily level similarity. Matchprot also detected some similarities in structures which are not in classified in the same SCOP fold (currently reported as false positives). Biological significance of these similarities are under study, and will be reported elsewhere. For detecting fold level similarity, we recommend a Z-score cuto3 value of 5. Also, Z-score increases with the average size of the structures. Thus, appropriate adjustments should be made in case of extremely low or high structure sizes.

Recently, CE [8] was rated to be the top fold recognition server [18]. Table 5 compares the effectiveness of Matchprot and CE for fold recognition purpose. We used a CE Z-score cuto3 of 4.0 as recommended for detecting hits. For both the programs, number of structures correctly detected, no. of false positives detected, precision and recall are reported. Precision and recall are defined as:



It can be seen that Matchprot performs favorably with CE. Thus, Matchprot is found to be effective for retrieving proteins having similar folds.

### Timing comparison

**Figure 2:**
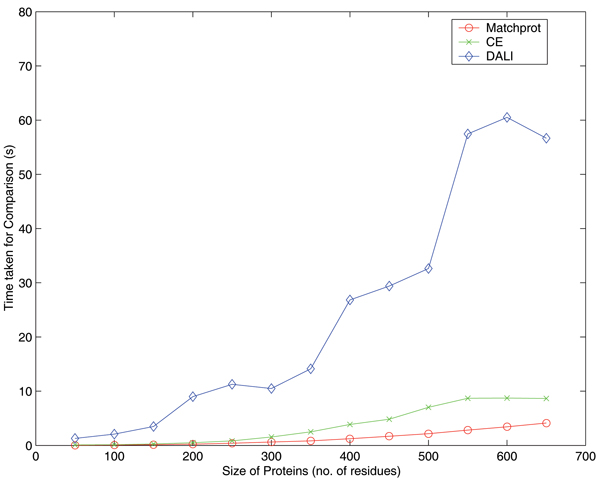
Comparison of time taken by CE, Matchprot and Dali for different sizes of structures.

Matchprot was designed for fast comparison of protein structures. In this section, we compare the time taken by Matchprot with those taken by CE and Dali. Each of the programs was run 30 times on protein structures for each size. The homologous structure was created from the parent structure by perturbing it randomly. The average time taken the programs for each size is reported in fig. 2. The sizes were varied from 50 to 650 in steps of 50. All the experiments were run on an Intel Pentium4 2.4 GHz machine. Also, in the experiment with Fischer's dataset, Matchprot was run for 72 combinations of parameters for 68 protein pairs, making a total of 4896 pairwise comparisons. Total time (user + system) taken by Matchprot was 27 min 38.58 s on a 2.4 GHz machine. Dali took 52 min 8.21 s for the same 68 protein pairs. The results in figure 2 demonstrate that Matchprot is 15–20 times faster than Dali. Moreover, it is 2–3 time faster than CE, which a well known database search program. Thus, Matchprot can used efficiently for searching databases of proteins.

## Conclusion

In this article, we describe a new protein structure retrieval algorithm called Matchprot. The algorithm was first validated on two comprehensive benchmark datasets against the standard protein structure comparison algorithm Dali. Performance of Matchprot was shown to be competitive with DALI on the benchmark. Next, it was shown that Matchprot can successfully detect non-topological similarities between two protein which is missed by DALI. Further validation was performed with proteins having multiple domains and internal repeats. Comparison of multi-domain proteins with individual domains showed that Matchprot is able to detect correct similarities in presence of up to 40% indels.

As a retrieval tool, Matchprot was successfully used to search SCOP 95% non-redundant dataset, having nearly 10000 protein structures, for 5 diverse types of proteins. The results showed encouraging accuracy when validated against SCOP. Matchprot was found competitive the standard database searching algorithm CE, in terms of search accuracy. Finally, the previous section shows that Matchprot outperforms state of the art programs in terms of speed.

The key to the speed of Matchprot is the a similarity function, based entirely on the structures, which measure the structural similarity between 2 residues, one from each structure. Such a function is designed using a novel characterization of residues of a structure based on projections that try to preserve neighborhoods. This characterization also connects to the spectral graph theoretic techniques popular in many disciplines. Optimal equivalences are calculated using a greedy fragment pair search heuristic. The algorithm has a running time of *O*(*n*^3^).

The main drawback of the current algorithm is it's inability to perform in the presence of a large number of indels. Thus, it needs to be made more robust toward indels. Though a small amount of indels (about 20%) are tolerated by the algorithm, in the case of multiple domains, only the largest one is detected. A work arround this problem could be to search through a domain database, e.g. ASTRAL, instead of the PDB, and decompose the query into constituent domains. Theoretical bounds on performance is another desirable development. Since, the problem of comparing protein structures is NP-hard, approximations having a comprehensive theory behind them are highly desirable.

Empirical results show that Matchprot is capable of detecting non-topological similarities (circular permutations in particular) between two proteins. Circular permutations are connected to many important questions in molecular biology. A study of suitability of Matchprot for automatic detection circularly permuted pairs of proteins is underway and will be reported elsewhere.

## Methods

### Problem description

A protein is a polymer of amino acids (also called residues). The protein sequence is a string comprising of the type of residues arranged in the order they are connected in the polymer. The protein structure is described by the 3D coordinates of all non-hydrogen atoms present in the protein. However, following the common practice we will use the coordinates of the *C*^*α *^atoms of the residues to describe each protein structure. Thus, a protein structure having *m *residues will be given by a set of points *A *= {*x*_1_, *x*_2_,...,*x*_*m*_),**x**_*i *_∈ ℝ ^3^, 1 ≤ i ≤ m, where each point represents one residue. The sequence ordering of the residues of the protein is given by the indices of the points.

A *structural alignment *between two protein structures *A *and *B *is given by a set of *equivalences *(1 – 1 correspondences) between the residues of the 2 proteins. So, a structural alignment *Φ *between structures *A *and *B*, of length *L*, is denoted as:

*Φ*(*A, B*) = {(*i*_*l*_, *j*_*l*_)|1 ≤ *l *≤ *L*, 1 ≤ *i*_*l *_≤ m, 1 ≤ *j*_*l *_≤ n, and *i*_*l *_= *i*_*k *_or *j*_*l *_= *j*_*k *_iff *l *= *k*}. In the alignment the  residue of protein A is said to be matched or equivalenced with the *j*_*l*_^*th *^residue of protein *B*.

**Figure 3:**
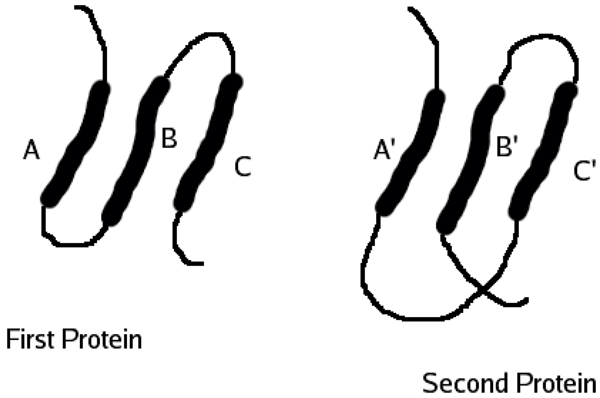
Non-topological similarity between 2 proteins. Sequence ordering of the first protein is **A-B-C and that of the second protein is A'-C'-B'.**

Intuitively, an alignment is said to be good if the matched residues could be superposed on each other using a rigid transformation. This is captured by the most commonly used measure of similarity called Root Mean Square Deviation (RMSD), defined as *RMSD *= , where  is the optimal transformation. However, small RMSDs can be obtained by choosing a very small no. of matched residues, which can be superposed tightly. Thus, the no. of matched residues (also called length of alignment) is also an important parameter. Detection of non-topological similarities increases the length of an alignment while keeping the RMSD same. For example, in figure 3, fragments A, B, and C of the first protein could be superposed with the fragments A', B', and C' of the second protein respectively. However, an algorithm incapable of detecting non-topological similarities will only matches of fragments A, B with A', B' or A, C with A', C'.

Minimization of RMSD has been the objective of many methods including those described in [6,21], etc. However, many other formulation have been described in literature. Two other popular formulations are distance matrix overlap [3,4] and contact map overlap [22] problems, both of them being NP-Hard. The distance matrix overlap formulation involves finding permutations of rows and columns of the distance matrices of the 2 structures, so that the corresponding pairwise distances are roughly the same. Solution to this problem has been attempted in Dali [3] using Monte Carlo optimization. Combinatorial Extension (CE) [8] uses heuristic cutoffs on pairwise distance scores to develop a fast algorithm. Solution of the contact map overlap problem has been attempted using Integer Programming and Lagrangian relaxation method in [23]. A review of the methods is available in [24]. The problem can also be viewed as a weighted maximum common subgraph problem.

Next section discusses the *distance matrix *formulation of the problem [3], and motivates a new scoring function. The distance matrix **d**, is defined as: *d*_*ij *_= ||**x**_*i *_- **x**_*j*_||_2_. We will denote the *i*, *j*^*th *^entry of the distance matrix of structure *A *by  Frobenius norm is defined as ||**A**||*F *= .

### Motivation from Dali score function

To derive intuitions for the proposed formulation, it is instructive to understand what alignments Dali [3,4] might prefer. Dali defines the score of an alignment *Φ*(*A, B*) as follows



where  = ( + )/2. Dali searches the space of all feasible alignments for the *Φ *which maximizes *S*_*Dali*_.

There are two properties that make terms in the Dali scoring function (1) contribute high values to the total score. The first point to be noted is that residues which are spatially far do not contribute much to the Dali score. See that whenever residues *i*_*l*_, *i*_*k *_and *j*_*l*_, *j*_*k *_are far apart  is high, driving the exponential weighting term to 0. Hence spatially far residues have no impact on the score function. Dali thus considers only those residues which are spatially close, hence effectively defining a neighborhood.

The second observation is that Dali will try to match those residues which have a similar neighborhood. This is because the Dali score (1) function gives a higher score to those *Φ*'s, whose residues are such that | - | is low. Thus Dali tries to pick up alignments whose residues have similar spatial neighborhoods.

Searching for such alignments in the space of all possible alignments is an extremely difficult task. Dali uses interesting heuristics to solve the problem. However, as seen in the experiments, the solution is too slow for searching large databases of protein structures. The key to efficiently locating such alignments lies in utilizing the neighborhood information for characterizing the residues so that they can be compared readily. In the next section we discuss one such characterization.

### Optimally neighborhood preserving projections

As described earlier, a protein can be viewed a collection of points (residues) in ℝ^3^. We propose to assign real numbers (called *projections*) to these points satisfying two criteria. Firstly, points which are close to each other in the original structure should have projection values close to each other. Secondly, the projection values should be directly comparable across structures. Note that the first criterion comes from the study of Dali score function in the previous section. The second criterion is imposed to aid the design of a novel similarity function which will be useful later. In this section we discuss a formulation to optimally compute such projections.

To capture the notion of closeness we define the *nearness matrix * of a protein as the following nonlinear decreasing function of the distance matrix:



where, *d*_*i*,*j *_is the distance between *i*^*th *^and *i*^*th *^residues, *d*_*ij *_= ||*x*_*i *_- *x*_*j*_||_2_. The parameter a governs the rate of decrease of the nearness value.

Let **f **= [*f*_1_, ..., *f*_*n*_]^*T *^be the vector of projections of a protein having *n *residues. We require that whenever ^*ij *^is high, |*f*_*i *_- *f*_*j*_| should be low. This can be directly translated as the objective function:

.

However, this problem has a trivial solution, i.e., **f **= *c***e**, **e **= [1,..., 1]^*T *^∈ ℝ^*n*^. Instead, consider the problem:



which is same as: **f**^*T *^**f**.

However, this objective function has an unbounded solution. So, we fix the norm of **f**, to some constant value *c*.



For a given *c*, solving (4) is equivalent to finding the eigenvector corresponding to the maximum eigenvalue of , normalized to *c*^2^. This formulation essentially tries to keep |*f*_*i *_+ *f*_*j*_| high in addition to keeping |*f*_*i *_- *f*_*j*_| low, for *i*, *j *for which _*ij *_is high. The optimal solution to (4), is the *optimally neighborhood preserving projection *of a protein with nearness matrix .

Given two proteins, *A *and *B*, consisting of *m *and *n *residues, the second criterion requires that the respective projections **f**^*A *^and **f**^*B *^be comparable. We use this criterion to fix values for *c*^*A *^and *c*^*B*^. To do this, one can impose various criteria. We require the mean squared distance of the projection values of two proteins, from the origin to be equal, i.e.,

Thus, we have (*c*^*A*^)^2 ^= *m *and (*c*^*B*^)^2 ^= *n*. Our final optimization problem for calculating comparable, optimally neighborhood preserving projections becomes:



As noted earlier, this problem is equivalent to finding the eigenvector corresponding to the largest eigenvalue of , a problem that can be solved in *O*(*n*^2^) time [25]. In the next section, we give an alternative interpretation of this formulation followed by a generalization.

### 0.1 *k*-mutually orthogonal projections

In the previous section, we have described a formulation for computing projections of residues that optimally preserve the neighborhoods. It turned out to be same as computing the eigenvector corresponding to maximum eigenvalue of the nearness matrix, and assigning it's components to the corresponding residues.

A structural alignment is a 1–1 map between subsets of residues of the 2 proteins, or equivalently, a 1–1 map between subsets of basis directions of the space in which the projection vectors are embedded. From the distance matrix overlap formulation [3], we can also view a structural alignment as a 1–1 map from a subset of the basis of one distance matrix to that of the other distance matrix. Since, we use an invertible function to calculate the nearness matrices from distance matrices, the same formulation holds true for nearness matrices as well. The only difference being that, as intended, lower distances will have more significant values.

From the spectral decomposition of square symmetric matrices having full rank, we know that,  where *ζ*_i _is the *i*^*th *^eigenvector of  normalized to 1. For convenience, arrange *λ*_*i*_s and *ζ*_*i*_s such that *λ*_*i *_≥ *λ*_*j *_if *i *<*j*.

If ^*A *^and ^*B *^are nearness matrices of two similar proteins, they will have a common similar sub-matrix under a permutation of their bases. Considering only the two sub-matrices, and only the first term on the right hand side of the above equation, it is clear that the corresponding sub-vectors of  and  will have similar values under the same permutation of bases, as they are the same eigenvectors of similar matrices.

Under the assumption that the eigenvector entries for equivalenced residues are not significantly perturbed by inclusion of other entries in the matrix (we will come back to this later in the article), we can compare entries of the first eigenvector for finding the best permutation (or equivalences).

We make amendments for unequal number of residues in the two proteins by normalizing the eigenvectors to the number of residues in each protein. Explanation for the choice of eigenvector corresponding to the maximum eigenvalue can be given using the equation:



It can be seen that the error in reconstruction (*η*) of  is minimized if the values of *λ*_*j*_s on the RHS of eqn (6) are low. So we arrive at the same formulation of the characterization as in eqn (5).

We can generalize the formulation by requiring *k *mutually orthogonal projections instead of just one. We are looking for *k *vectors **f**^1^, · · · , **f**^*k*^, such that



In order to maintain the property of being comparable across structures, we normalize all the eigenvectors to the number of residues. Thus, each residue *i *of a protein is now characterized by a *k*-component projection vector say **p**_*i*_, calculated as *p*_*i*_(*j*) = , *i *= {1, · · · , *n*}, *j *= {1, · · · , *k*}. Experimental results on random matrices show that about 20% and 40% error in incurred by choosing *k *to be 3 and 1 respectively. Empirically, it is seen that not much accuracy is gained by chosing *k *greater than 3. In the next section, we use this characterization of the residues to define a similarity function between them.

### A measure of similarity

The problem we encounter while defining a similarity function using the characterization of the residues obtained in the previous section is that the solution to eqn (7) is not unique. It can be easily seen that if **f **is a normalized eigenvector of a matrix, then so is -**f**. Thus the solutions of eqn (7) for two proteins can not be compared directly.

There can be many ways of taking care of this ambiguity in representation. For example, we could have compared the absolute values of the projections. However, this has less discriminative power due to the loss of sign. Another way is to consider all 2^*k *^combinations of ± **f**^*j*^, *j *= 1, · · · , *k *and choose the best. The characterization of residues in the *k*-dimensional projection space, as derived the previous two sections can be compared in various ways. We consider the norm of difference of projections of 2 residues, a measure of distance between them. Thus, we define the similarity between residue *i *from protein *A *and residue *j *from protein *B *as:



where *T *(threshold) is a parameter and *p*_*i*_(*j*) = , *i *= {1, · · · , *n*}, *j *= {1, · · · , *k*}.

The most important feature of the above similarity measure is that it can score 2 residues one from each structure based on purely structural properties (no information about the protein sequence or secondary structural elements have been used). Secondly, this similarity measure gives a similarity between 2 residues, one from each structure, as opposed to the typical ones which take 4 residues, 2 from each structure (e.g. Dali [3] and CE [8]). Such a similarity function can be used to design very fast algorithms, and is the key to the speed of Matchprot.

The above formulation works perfectly when there are no extra residues (indels) in the two proteins. However, if there are a large number of indels between the structures, the projections of the residues participating in the alignment are likely to be disturbed by the extra residues, thereby giving incorrect equivalences. We have also investigated the maximum percentage of indels that are tolerated. In the next section, we describe a method for finding the optimal equivalences from the above derived similarity function.

### Finding optimal equivalences

Given an alignment *Φ *= {(*i*_*l*_, *j*_*l*_)|1 ≤ *l *≤ *L*}, and a scoring function of the form *s*(*i*_*l*_, *j*_*l*_), an obvious measure of goodness of *Φ *is:



The problem of finding alignment that maximizes the objective function (9), can be posed as an assignment problem and solved exactly. However, the solution will be slow and will not have any relation to the protein sequence.

On the other hand, one can compute a structural alignment between two protein structures by globally aligning the sequences of the two proteins using *s*(*i*, *j*) as the score function. However, this will not capture the non-topological similarities between the two proteins.

We propose to greedily pick common subsequences with high structural similarity. The problem can be posed as that of finding two subsequences which have maximum similarity with the pairwise similarity score given by eqn (8). This is same as the *Local Alignment problem *[26] and can be solved efficiently using dynamic programming. We call such pairs of subsequences as *High Scoring Fragment Pairs (HSFPs)*. The algorithm operates in 2 stages:

(1) Calculation of the local alignment matrix with similarity score given by equation 4.

(2) Iterative determination of HSFPs and their elimination from the local alignment matrix.

The local alignment matrix *L*, is computed as:



where, *g *is the gap penalty provided as a parameter to the program. The highest scoring entry (corresponding to highest scoring fragment pair) is detected and traced back to get the highest scoring fragment. The indices corresponding to the residues participating in the current alignment are eliminated from the matrix and the above step is repeated to get the next highest scoring fragment. This is stopped when there are no more positive scoring fragments. The steps are given in Algorithm 1.

**Algorithm 1 **Finding Equivalences

1: *Alignment *← *φ*.

2: Compute *highest *= *max*_*i*,*j *_*L*_*i*,*j*_.

3: Compute (*u*, *v*) = *arg *max_*i*,*j *_*L*_*i*,*j*_. {*u *and *v *are the residues indices}

4: **while ***highest *> 0 **do**

5: *Alignment ← Alignment ∪ traceback *(*u, v*) {*traceback *returns the alignment obtained by tracing back from it's argument}

6: Mark the rows and columns of *L *corresponding to the residues returned in the current alignment *done*.

7: Compute *highest *= *max*_*i*,*j *_*L*_*i*,*j *_such that *i *or *j *is not marked *done*.

8: Compute (*u, v*) = *arg *max_*i*,*j *_*L*_*i*,*j *_such that *i *or *j *is not marked *done*.

9: **end while**

Finally, the fragment pairs found above are concatenated to get the whole alignment. To get the equivalences, all the residues aligned to gaps are discarded, and matching residue pairs are taken as equivalenced residues.

Computation of the (*m *+ 1) × (*n *+ 1) entries of the local alignment matrix takes *O*(*mn*) time. Detection of alignment fragments is done by searching through the (*m *+ 1) × (*n *+ 1) matrix for at most *min*(*m, n*) times, which takes *O*(*min*(*m*^2^*n*, *mn*^2^) time. This computation is repeated for 2^*k *^times, thus making the overall time complexity of the algorithm to be *O*(2^*k*^*max*(*m*^3^, *n*^3^)). For the current algorithm, *k *is taken to be very small thus making it *O*(*n*^3^). At any point of time, the program stores a constant number of *m *× *m*, *n *× *n *and *m *× *n *matrices. Thus, it consumes *O*(*max*(*m*^2^, *n*^2^)) memory space.

### Superposition, RMSD and statistical significance

From the equivalences, we can compute the rigid transformation of one structure into the other that minimizes the RMSD between the matching residues, using the method by Horn [27]. The optimal transformation can then be applied to the appropriate structure to compute the superposition. Once the optimal superposition has been computed, the similarity score is recomputed as *s*(*i*, *j*) = 5.0 - ||*X_i _*- *Y_j_*||. The final equivalences are calculated using Algorithm 1. This post-processing helps to keep the total RMSD low and also corrects some minor shifts in equivalences. We compute the Dali Z-score [28] to give a measure of the statistical significance of the alignment.

## Authors' contributions

S.B. and C.B. devised the algorithm; S.B. implemented it. N.C. helped in analyzing the results and interpreting the biological implications.

## Figures and Tables

**Table 1:** Comparison of results from Matchprot and Dali using Fischer's and Novotny's Benchmark Dataset.

Data set/Classifn.	Total pairs	Better	Worse	Level
Fischer Novotny et. al.	68	17	18	33
1.10.40	21	8	1	12
1.10.164	10	2	0	8
1.25.30	21	3	0	18
2.30 110	6	1	2	3
2.40.100	28	4	3	21
2.100.10	15	5	4	6
3.10.70	10	0	2	8
3.40.91	6	6	0	0
3.70.10	15	1	3	11
2.40.20	21	1	4	16

**Table 2:** Results from comparison of multi-domain proteins with partial structures and individual domains.

ID1 – ID2	No. of Deletions/Total Size	Matchprot (Lali/RMSD/Zscore)	Dali (Lali/RMSD/Zscore)
2hcka – 2hcka-4	4/437	433/0.00/91.60	434/0.00/60.5
2hcka – 2hcka-8	8/437	429/0.00/91.21	430/0.00/60.5
2hcka – 2hcka-12	12/437	425/0.00/90.85	426/0.00/60.5
2hcka – 2hcka-20	20/437	417/0.00/90.75	418/0.00/60.3
2hcka – 2hcka-50	50/437	387/0.00/93.63	388/0.00/60.5
2hcka – 2hcka-100	100/437	337/0.00/76.49	338/0.00/49.3

2hcka – d2hcka1	374/437	34/2.81/-1.18	63/0.0/15.1
2hcka – d2hcka2	334/437	59/3.12/-0.63	103/0.0/21.6
2hcka – d2hcka3	166/437	271/0.00/58.53	272/0.0/43.3
2src – d2src_1	387/449	45/3.33/-1.56	62/0.0/15.1
2src – d2src_2	346/449	68/3.31/-0.83	103/0.0/22.9
2src – d2src_3	165/449	284/0.00/64.61	285/0.0/46.4

**Table 3:** Results from comparison of proteins with internal repeats.

PDB1 – PDB2	Matchprot (Lali/RMSD/Zscore)	Dali (Lali/RMSD/Zscore)
1gyhA – 1tl2A	179/3.36/7.79	196/3.9/7.4
1nscA – 3sil	291/2.99/37.52	289/3.2/23.5
1bd8 – 1ihbA	154/1.26/27.99	154/1.3/25.2
1l4aA – 1n7sA	62/1.58/5.61	62/1.7/5.6
2pec – 1bn8A	282/2.07/49.15	287/2.5/32.1
1kapP – 1sat	444/1.43/70.71	448/1.7/49.9

**Table 4:** Detection of Similar Proteins by Matchprot.

Query ID (SCOP classfn.)	SCOP sim. level	Z-Score cutoff	No. of structures (actual/detected/false +ve)
d101m__	Family	12	64/64/0
(a.1.1.2)	Superfamily	5	93/93/0
	Fold	5	97/93/0
d1htia_	Family	20	15/15/0
(c.1.1.1)	Superfamily	20	15/15/0
	Fold	6	327/272/56
d1jzba_	Family	4	17/17/0
(g.3.7.1)	Superfamily	2	55/23/0
	Fold	2	238/23/0
d2pela_	Family	25	26/26/0
(b.29.1.1)	Superfamily	5	87/70/50
	Fold	5	87/70/50
d7rsa__	Family	5	18/18/0
(d.5.1.1)	Superfamily	5	18/18/0
	Fold	5	18/18/0

**Table 5:** Comparison of results for SCOP database search from Matchprot with those from CE.

Query ID	Matchprot (detected/false +ve/precision/recall)	CE (detected/false +ve/precision/recall)
d101m__	93/0/1/0.95	96/2/0.97/0.99
d1htia_	272/56/0.82/0.83	307/29/0.91/0.93
d1jzba_	23/0/1/0.1	33/270/0.1/0.14
d2pela_	70/50/0.58/0.8	61/36/0.62/0.70
d7rsa__	18/0/1/1	17/1/0.94/0.94

## References

[B1] Berman HM, Westbrook J, Feng Z, Gilliland G, Bhat TN, Weissig H, Shindyalov IN, Bourne PE (2000). The Protein Data Bank. Nucleic Acids Research.

[B2] Goldman D, Papadimitriou CH, Istrail S (1999). Algorithmic Aspects of Protein Structure Similarity. FOCS '99: Proceedings of the 40th Annual Symposium on Foundations of Computer Science.

[B3] Holm L, Sander C (1993). Protein Structure Comparison by Alignment of Distance Matrices. Journal of Molecular Biology.

[B4] Holm L, Sander C (1996). Mapping the Protein Universe. Science.

[B5] Bachar O, Fischer D, Nussinov R, Wolfson H (1993). A Computer Vision Based Technique for 3-D Sequence Independent Structural Comparison of Proteins. Protein Engineering.

[B6] Singh AP, Brutlag DL (1997). Hierarchical protein structure superposition using both secondary structure and atomic representations. Proceedings of International Conference on Intelligent Systems in Molecular Biology.

[B7] Taylor WR, Orengo CA (1989). Protein Structure Alignment. Journal of Molecular Biology.

[B8] Bourne PE, Shindyalov IN (1998). Protein structure alignment by incremental combinatorial extension of optimal path. Protein Engineering.

[B9] Lindqvist Y, Schneider G (1997). Circular permutations of natural protein sequences: structural evidence. Current Opinion in Structural Biology.

[B10] Uliel S, Fliess A, Unger R (2001). Naturally occuring circular permutations in proteins. Protein Engineering.

[B11] Jung J, Lee B (2001). Circularly permuted proteins in the protein structure database. Protein Science.

[B12] Shih ES, Hwang MJ (2004). Alternative Alignments from Comparison of Protein Structures. PROTEINS: Structure, Function, and Bioinformatics.

[B13] Umeyama S (1988). An eigendecomposition approach to weighted graph matching problems. IEEE transactions on pattern analysis and machine intelligence.

[B14] Kawabata T, Nishikawa K (2000). Protein Structure Comparison Using the Markov Transition Model of Evolution. Proteins.

[B15] Anderson E, Bai Z, Bischof C, Blackford S, Demmel J, Dongarra J, Du Croz J, Greenbaum A, Hammarling S, McKenney A, Sorensen D (1999). LAPACK Users' Guide.

[B16] Rasmol: Molecular Graphics Visualisation Tool. http://www.openrasmol.org.

[B17] Fischer D, Elofsson A, Rice DW, Eisenberg D (1996). Assessing the Performance of Fold Recognition Methods By Means of a Comprehensive Benchmark. Pacific Symp on Biocomputing.

[B18] Novotny M, Madsen D, Kleywegt GJ (2004). Evaluation of Protein Fold Comparison Servers. Proteins.

[B19] Murzin AG, Brenner SE, Hubbard T, Chothia C (1995). SCOP: a structural classification of proteins database for the investigation of sequences and structures. Journal of Molecular Biology.

[B20] Chandonia JM, Hon G, Walker NS, Conte LL, Koehl P, Levitt M, Brenner SE (2004). The ASTRAL compendium in 2004. Nucleic Acids Research.

[B21] Kolodny R, Linial N (2004). Approximate protein structural alignment in polynomial time. Proc Natl Acad SciUSA.

[B22] Godzik A, Skolnick J (1994). Flexible algorithm for direct multiple alignment of protein structures and sequences. CABIOS.

[B23] Caprara A, Carr R, Istrail S, Lancia G, Walenz B (2004). 1001 Optimal PDB Structure Alignments: Integer Programming Methods for Finding the Maximum Contact Map Overlap. Journal of Computational Biology.

[B24] Eidhammer I, Jonassen I, Taylor WR (2000). Structure Comparison and Structure Patterns. Journal of Computational Biology.

[B25] Dhillon IS (1997). A new *O*(*N*^2^) algorithm for the symmetric tridiagonal eigenvalue/eigenvector problem.

[B26] Smith T, Waterman M (1981). The identification of common molecular subsequences. Journal of Molecular Biology.

[B27] Horn BKP (1987). Closed form solution of absolute orientation using unit quaternions. Journal of the Optical Society of America.

[B28] Holm L, Sander C (1998). Dictionary of recurrent domains in protein structures. Proteins.

